# Wheat and Bread

**Published:** 1872-10

**Authors:** 


### WHEAT AND BREAD.

One fifth of the wheat crop is lost to the world every year for purposes of human aliment. It is lost in the bran, and is fed to the animals; it is because the nutriment sticks so closely to the bran, which is the skin of the wheat-grain, that it cannot be separated by any means yet known. The only plan is either to get the skin off before the wheat is ground, or to grind all together, and eat it all together, without attempting to remove the bran; this makes Graham bread, bread made of the whole of the wheat-grain. If children, until the age of ten years, were made to take this alone with milk, for their breakfast, it would give them perfect teeth, hard, white, and enduring; their bones would be stronger, their flesh firmer, and their general constitutions so much improved, that there would be a comparative exemption from the ordinary diseases of childhood. It would make our children like the Scotch, who so largely live on oat-meal, which is made of oats ground up into coarse flour, and converted into porridge. In a forthcoming Journal, a method will be proposed for taking the skin from the wheat perfectly, at little cost, at the rate of five hundred bushels an hour, taking off the bran alone, without a particle of the nourishment of the wheat. If this is successful on a large scale, as it has been on a small one, it will be equivalent to adding one fifth to the wheat crop of the world, as human food. If so, it is the most important discovery of the age in reference to what we eat.

Meanwhile, inventors are working in another direction, devising a method of preventing the destruction of wheat and corn and other grain, by "heating," on its way from the farm to the warehouse of the merchant, to the mill, or to a foreign port. It is a subject of immense importance to every farmer directly, and to every man, woman, and child in the land, because, in a country like ours, where we are obliged to export a large share of the grain raised, on account of our inability to consume it, the foreign market controls the price. Therefore the farmer, instead of fixing the price, is obliged to take the rate fixed by the foreign market; or, in other words, what may be left after paying transportation, commissions, insurance, and a percentage of risk on the damage to the grain while in transit. This latter point is not commonly understood, but is nevertheless true, as all merchants charge a certain sum for every risk taken. Large quantities of grain are greatly impaired, or rendered almost valueless, every year, by reason of "heating;" and when this process begins, unless immediately arrested, the destruction is complete within a few days. Hence it is seen how important a subject is the treating of grain in a suitable and economical manner for transportation; for as the loss by heating enters into the calculation of cost, in disposing of the grain, and as the foreign market practically controls the price of the home market, it will be seen what an enormous sum of money is annually lost to the farmer, to say nothing of the anxiety of the shipper.

Objections have been made to drying or curing grain, because the moisture evaporated lessened the weight; while this is true, no actual loss is made, for when the grain is ground, it will absorb the more water in making it into bread.

These remarks are suggested by seeing in our office a sample of corn treated by a new process, which will either partially dry or kiln-dry the grain, completely destroy all insect life, and thoroughly disinfect and free it of every offensive odor. The cost of apparatus which will treat five hundred bushels an hour cannot exceed three thousand dollars, while it is extremely simple, easily controlled, occupies but little space, and has the least possible liability to get out of repair; the expense of working would be a small fraction of a cent per bushel.

It is believed that this invention will revolutionize the handling of grain, and will save millions of bushels of it for purposes of human food, which are now lost annually.

